# NRF2 Drives Aggressiveness and Chemoresistance in Ovarian Cancer Stem-like Cells

**DOI:** 10.3390/ijms27062820

**Published:** 2026-03-20

**Authors:** Yu-Hsun Chang, Kai-Hung Wang, Dah-Ching Ding

**Affiliations:** 1 Department of Pediatrics, Hualien Tzu Chi Hospital, Buddhist Tzu Chi Medical Foundation, Tzu Chi University, Hualien 970, Taiwan; cyh0515@tzuchi.com.tw; 2 Department of Medical Research, Hualien Tzu Chi Hospital, Buddhist Tzu Chi Medical Foundation, Tzu Chi University, Hualien 970, Taiwan; kennyhug0201@tzuchi.com.tw; 3 Department of Obstetrics and Gynecology, Hualien Tzu Chi Hospital, Buddhist Tzu Chi Medical Foundation, Tzu Chi University, Hualien 970, Taiwan; 4 Institute of Medical Sciences, Tzu Chi University, Hualien 970, Taiwan

**Keywords:** NRF2, ovarian cancer, cancer stem-like cells, chemoresistance, oxidative stress signaling

## Abstract

Advanced-stage ovarian cancer remains a major clinical challenge because of its aggressive behavior and the frequent development of chemoresistance. The nuclear factor erythroid-derived 2–like 2 (NRF2) signaling pathway regulates cellular redox homeostasis. However, its role in ovarian cancer stem-like cells remains unclear. Therefore, we aimed to investigate the effects of NRF2 overexpression on acetaldehyde dehydrogenase (ALDH)^+^ KURAMOCHI ovarian cancer cells in vitro and in vivo. In particular, we investigated the effects of NRF2 on tumor-associated behaviors, chemoresistance, and signaling pathways. Lentivirus-mediated NRF2 overexpression activated extracellular signal-regulated kinase and AKT signaling. Moreover, it modulated tumor-associated phenotypes, including proliferation, migration, and invasion. NRF2-overexpressing cells exhibited significantly enhanced migratory and invasive capacities, increased resistance to paclitaxel and carboplatin, and reduced apoptosis. Furthermore, the expression of anti-apoptotic proteins was upregulated, and caspase-3 activation was attenuated. In xenograft models, NRF2 overexpression promoted tumor growth and increased the expression of antioxidant and angiogenic factors, including heme oxygenase-1 and vascular endothelial growth factor A. Collectively, these findings demonstrate that NRF2 regulates ovarian cancer aggressiveness and chemoresistance by coordinating stress response signaling, survival pathways, and tumor progression. Therefore, targeting NRF2-mediated signaling represents a promising therapeutic strategy for overcoming drug resistance and improving outcomes in patients with ovarian cancer.

## 1. Introduction

Among gynecological cancers, ovarian cancer causes the most deaths annually. An estimated 21,750 new cases and 13,490 deaths have occurred in the United States [1]. In Taiwan, 1677 new cases were diagnosed in 2019 (incidence: 9.9/100,000) and 696 deaths were reported in 2021 (according to the Ministry of Health and Welfare, Taiwan, https://www.mohw.gov.tw/cp-7177-82775-1.html (accessed on 30 January 2026). The most common form of ovarian cancer is the epithelial type, which is often diagnosed at advanced stages [2]. Current management includes debulking surgery, followed by adjuvant chemotherapy and/or targeted therapy [3]. Despite primary therapy, the recurrence rate is 70% within the first 5 years of diagnosis with FIGO stages III to IV [3]. Thus, significant improvements are required in the treatment of advanced-stage ovarian cancer [4,5].

Given the persistent therapeutic challenges and poor outcomes associated with advanced-stage ovarian cancer, increasing attention has been directed toward molecular pathways that regulate cellular stress responses and survival. Of these, the nuclear factor erythroid-derived 2–like 2 (NRF2) pathway plays a pivotal role in the protection of cells against oxidative damage induced by external and endogenous stressors [6]. This pathway helps maintain redox homeostasis, exerts anti-inflammatory and anticancer effects by regulating various downstream cytoprotective genes, and is vital for cell survival [6]. Recent evidence indicates that NRF2 plays contradictory roles in cancer [7]. Its aberrant activation is associated with poor prognosis in certain cancers [8]. In various cancer types, constitutive activation of NRF2 induces pro-survival genes, promotes cancer cell proliferation through metabolic reprogramming, suppresses cancer cell apoptosis, and enhances the self-renewal capacity of cancer stem cells [9]. Moreover, NRF2 contributes to the chemoresistance, radioresistance, and inflammation-induced carcinogenesis of cancer cells [10].

NRF2 plays a critical, multifaceted role in ovarian cancer development and progression [11]. Its aberrant activation promotes tumor initiation by enhancing cellular survival under oxidative stress, consequently enabling cancer cells to evade apoptosis and resist DNA damage [12,13]. In established disease, NRF2 contributes to chemoresistance by upregulating detoxification and drug efflux pathways, thereby reducing intracellular drug accumulation and diminishing the efficacy of chemotherapy [14]. Furthermore, sustained NRF2 signaling supports tumor progression by facilitating metabolic reprogramming, promoting proliferation, and enhancing invasive and metastatic potential through increased cytoprotective capacity [11]. Given these oncogenic functions, NRF2 represents an attractive therapeutic target. Pharmacological inhibition of NRF2 or its downstream pathways may improve chemosensitivity and limit tumor aggressiveness in ovarian cancer [15]. However, its exact role in ovarian cancer stem-like cells remains unclear.

In this study, we aimed to investigate the role of NRF2 in ovarian cancer stem-like cells and elucidate its effects on tumor-associated behaviors, chemoresistance, and underlying signaling pathways. This study further sought to determine whether NRF2 mediates ovarian cancer aggressiveness, and to evaluate its potential as a therapeutic target in the prevention of chemoresistance.

## 2. Results

### 2.1. NRF2 Overexpression Suppresses Proliferation and Increases ERK/AKT Signaling in KURAMOCHI Acetaldehyde Dehydrogenase (ALDH)^+^ Cells

**Figure 1:**
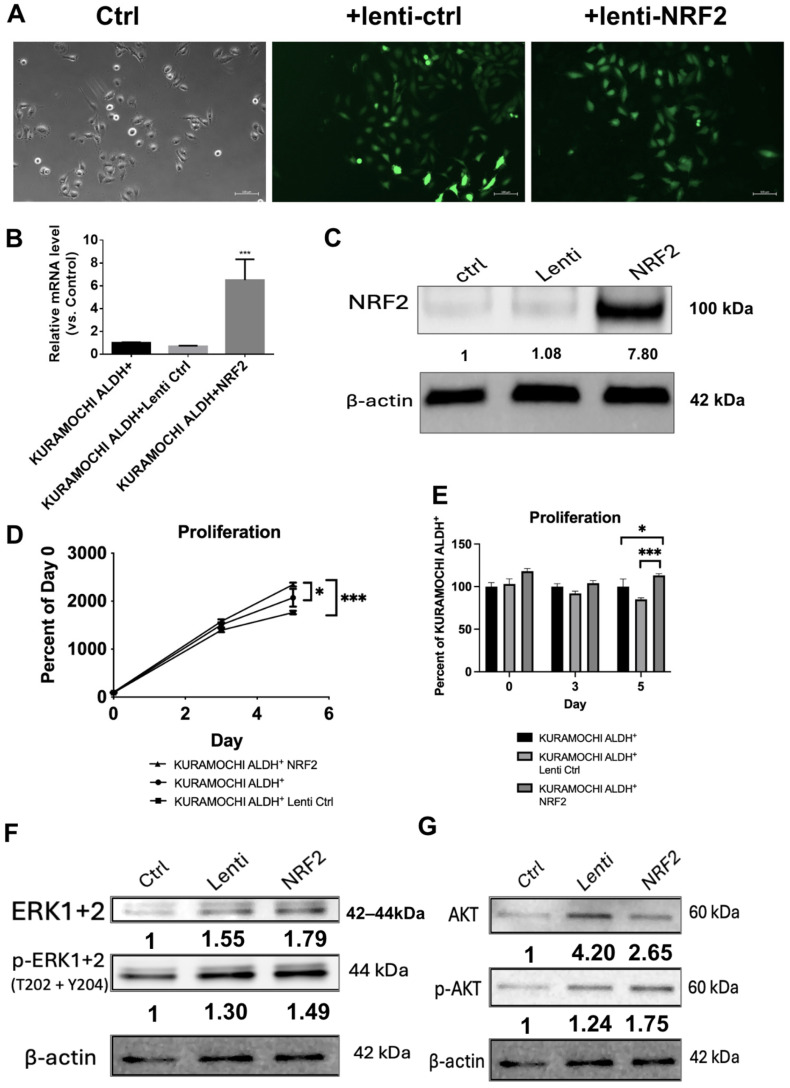
Overexpression of NRF2 in KURAMOCHI acetaldehyde dehydrogenase (ALDH)^+^ cells reduces proliferation and modulates signaling pathways. (**A**) Brightfield and fluorescence microscopy images confirming successful lentiviral transduction of NRF2 using GFP-tagged constructs in KURAMOCHI ALDH^+^ cells. Scale bar = 100 μm; (**B**) Quantitative RT-PCR analysis of NRF2 mRNA expression in the NRF2-overexpressing group compared to that in the control and lenti-control (*n* = 3, *** *p* < 0.001); (**C**) NRF2 protein levels in the NRF2-transduced group compared to those in the control group, normalized to β-actin (*n* = 1); (**D**) Cell proliferation assays of NRF2-overexpressing ALDH^+^ cells and the controls (*n* = 3; * *p* < 0.05, *** *p* < 0.001); (**E**) Proliferation of KURAMOCHI ALDH^+^ cells transduced with control lentivirus (Lenti Ctrl) or NRF2 overexpression vector over 5 days, expressed as a percentage relative to intact control cells, showing significantly increased proliferation in the NRF2 group at day 5 (* *p* < 0.05, *** *p* < 0.001). (**F**) Expression levels of phosphorylated ERK1/2 in the NRF2-overexpressing group, as determined by Western blotting (*n* = 1); (**G**) Expression levels of phosphorylated and total AKT in NRF2-overexpressing cells, as determined by Western blotting (*n* = 1).

KURAMOCHI ALDH^+^ cells were transduced with lentiviral vectors carrying NRF2 to investigate its function in ovarian cancer stem-like cells. Efficient transduction was confirmed by GFP fluorescence and increased NRF2 mRNA and protein levels (Figure 1A–C). NRF2 overexpression significantly increased cell proliferation compared to that in the control and vector-only groups (Figure 1D; *p* < 0.05 to < 0.001). NRF2 overexpression increased the proliferation of KURAMOCHI ALDH^+^ cells over time, with a significant increase observed at day 5 compared with the lenti-control group (Figure 1E). Western blot analysis revealed that NRF2 overexpression increased the phosphorylation of extracellular signal-regulated kinase (ERK) 1/2 and AKT without affecting their total protein levels (Figure 1F,G). This indicates that NRF2 promotes key oncogenic signaling pathways associated with cell growth. These data suggest that NRF2 functions as an oncogene in ovarian cancer stem-like cells by increasing ERK and AKT signaling.

### 2.2. NRF2 Overexpression Enhances Migration and Invasion

**Figure 2:**
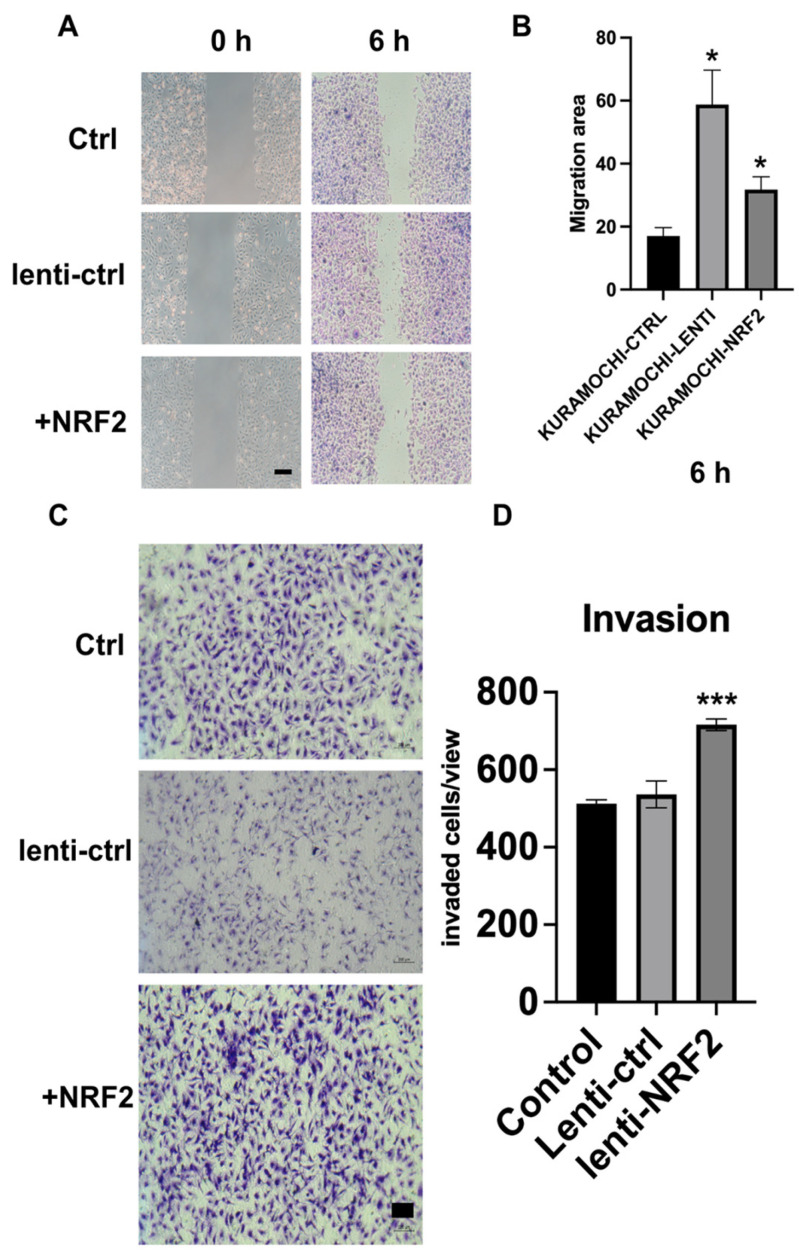
NRF2 overexpression enhances the migration and invasion of KURAMOCHI ALDH^+^ cells. (**A**) Representative images of wound-healing assays in KURAMOCHI ALDH^+^ cells under control (Ctrl), lentiviral control (lenti-ctrl), and NRF2 overexpression (+NRF2) conditions at 0 and 6 h. Wound closure was markedly accelerated in NRF2-overexpressing cells compared with controls. Scale bar = 100 μm. (**B**) Quantification of migration area at 6 h. NRF2-overexpressing cells exhibited significantly increased migration compared with Ctrl and lenti-ctrl groups (* *p* < 0.05). (**C**) Representative images of Transwell invasion assays showing invaded cells stained with crystal violet in Ctrl, lenti-ctrl, and NRF2-overexpressing cells. Increased invasive capacity was observed in the NRF2 group. Scale bar = 100 μm. (**D**) Quantification of invaded cells per field. NRF2 overexpression significantly enhanced invasion compared with control groups (*** *p* < 0.001). Data are presented as mean ± SD from at least three independent experiments. Scale bars as indicated.

As shown in Figure 2, NRF2 overexpression significantly enhanced the migratory and invasive capacities of KURAMOCHI ALDH^+^ ovarian cancer cells. In the wound-healing assay (Figure 2A,B), lenti-NRF2 cells exhibited markedly accelerated gap closure at 6 h compared to both the control and lenti-control groups. This was quantitatively confirmed by a significantly larger migration area. Consistently, 24 h Transwell invasion assays demonstrated a pronounced increase in the number of invaded cells in the NRF2-overexpressing group relative to the controls (Figure 2C,D). These findings indicate that NRF2 activation promotes aggressive phenotypes in ovarian cancer cells by enhancing both cell migration and invasion.

### 2.3. NRF2 Overexpression Confers Chemoresistance and Inhibits Apoptosis in KURAMOCHI ALDH^+^ Cells

**Figure 3:**
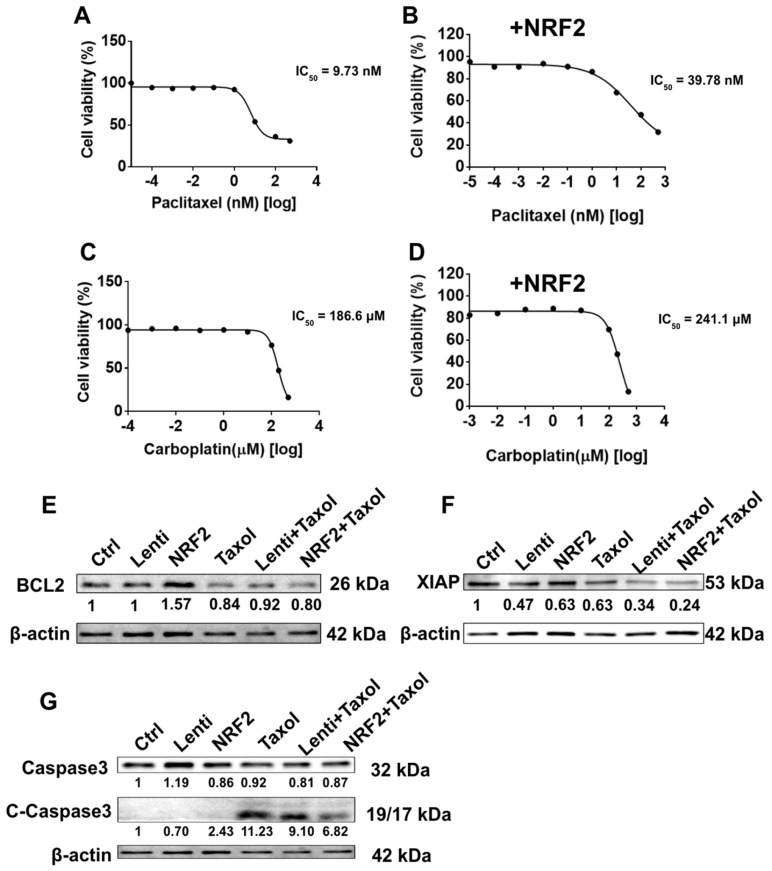
NRF2 overexpression reduces chemosensitivity to paclitaxel and carboplatin and modulates apoptosis-related proteins in KURAMOCHI ALDH^+^ cells. (**A**–**D**) Dose–response curves of paclitaxel and carboplatin in control (**A**,**C**) and NRF2-overexpressing (**B**,**D**) KURAMOCHI ALDH^+^ cells (*n* = 3). (**E**,**F**) Expression levels of BCL2 (**E**) and XIAP (**F**) following NRF2 overexpression alone or in combination with paclitaxel compared to the controls (*n* = 1), as determined by Western blotting. (**G**) Expression levels of caspase-3 in NRF2-overexpressing cells following paclitaxel treatment (*n* = 1).

Dose–response assays were conducted in KURAMOCHI ALDH^+^ cells to determine whether NRF2 affects chemosensitivity. NRF2 overexpression significantly increased the half-maximal inhibitory concentration (IC_50_) of paclitaxel from 9.73 nM to 39.78 nM and that of carboplatin from 186.6 μM to 241.1 μM (Figure 3A–D), indicating reduced sensitivity to both agents. Furthermore, NRF2 overexpression was associated with increased expression of the anti-apoptotic protein B cell lymphoma 2 (BCL2) (Figure 3E), whereas X-linked inhibitor of apoptosis protein (XIAP) expression was decreased in NRF2-overexpressing cells (Figure 3F). Contrastingly, the levels of cleaved caspase-3 were markedly reduced in NRF2-overexpressing cells treated with paclitaxel compared to those in the controls (Figure 3G). These findings suggest that NRF2 contributes to chemoresistance in ovarian cancer stem-like cells by upregulating survival signals and inhibiting caspase-dependent apoptosis.

### 2.4. NRF2 Overexpression Promotes Tumor Growth and Activates Oncogenic Signaling In Vivo

**Figure 4:**
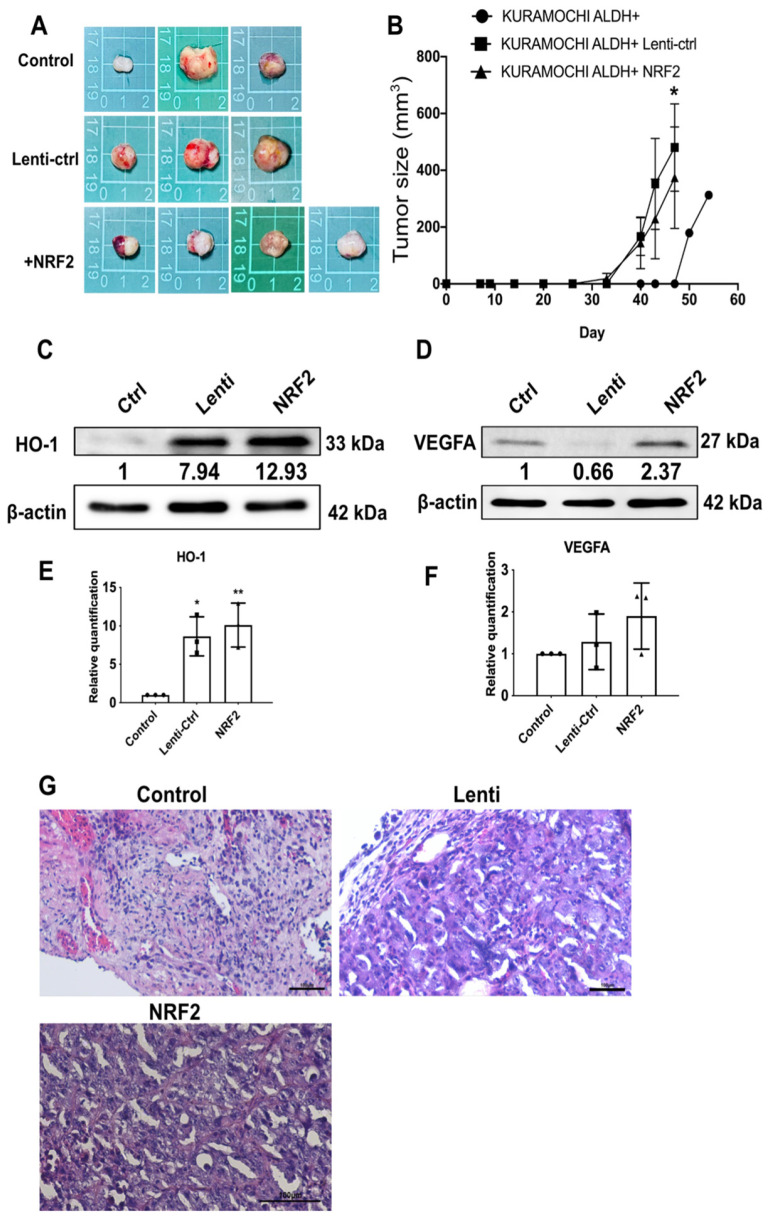
NRF2 suppresses tumor growth and modulates key oncogenic and antioxidant signaling pathways in vivo. (**A**,**B**) KURAMOCHI ALDH^+^ cells with or without NRF2 overexpression were subcutaneously injected into NOD-SCID mice. Representative tumor images (**A**) and tumor growth curves (**B**) demonstrating changes in tumor size in the NRF2-overexpressing group (*n* = 4) and leti-ctrl (*n* = 3) compared to controls (*n* = 3; * *p* < 0.05). (**C**,**D**) Representitive image of expression levels of the NRF2-regulated antioxidant protein HO-1 (*n* = 3) (**C**) and the angiogenic factor VEGFA (*n* = 3) (**D**) in xenograft-derived cells, as determined by Western blotting. (**E**,**F**) Relative quantification of HO-1 (**E**) and VEGFA (**F**) (*n* = 3). * *p* < 0.05, ** *p* < 0.01. (**G**) Representative hematoxylin and eosin (H&E) staining of xenograft tumor sections from Control, lentiviral control (Lenti), and NRF2-overexpressing groups. The NRF2-overexpressing tumors exhibited increased tumor cell density, enlarged and hyperchromatic nuclei, and more frequent mitotic figures compared with the Control and Lenti groups, indicating enhanced proliferative and malignant features. Scale bar = 100 μm.

KURAMOCHI ALDH^+^ cells with or without NRF2 overexpression were subcutaneously injected into non-obese, diabetic-severe combined immune deficiency (NOD-SCID) mice to assess the effects of NRF2 in vivo. Tumor formation was significantly increased in the NRF2 group, as evidenced by rapid tumor growth and increased tumor volumes compared to those in the control groups (Figure 4A,B; *p* < 0.05). Western blot analysis of xenograft tissues revealed increased expression of the antioxidant protein HO-1 (Figure 4C,E) and VEGFA (Figure 4D,F) following NRF2 overexpression. Hematoxylin and eosin staining of xenograft tumor sections revealed distinct histopathological differences among the groups (Figure 4G). Tumors derived from NRF2-overexpressing KURAMOCHI ALDH^+^ cells exhibited increased cellular density with more compact tumor architecture compared with the Control and Lenti groups. The NRF2 group also showed enlarged, hyperchromatic, and pleomorphic nuclei, along with more frequent mitotic figures, indicating increased proliferative activity. In contrast, the Control group demonstrated lower cellular density, more uniform nuclear morphology, and fewer mitotic figures, while the Lenti group displayed intermediate features (Table 1). These findings suggest that NRF2 overexpression is associated with enhanced tumor cell proliferation and increased malignant histological characteristics in vivo. These findings demonstrate that NRF2 promotes ovarian cancer stem-like cell tumorigenicity by modulating antioxidant defense and angiogenesis in vivo.

## 3. Discussion

**Figure 5:**
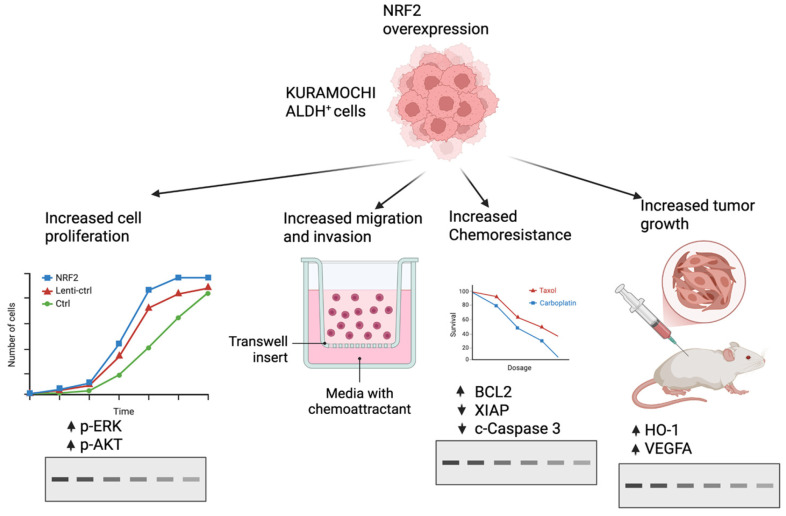
Schematic illustration showing that NRF2 overexpression in KURAMOCHI ALDH^+^ ovarian cancer stem-like cells promotes ERK/AKT activation, enhances proliferation, migration, invasion, and chemoresistance, and increases in vivo tumor growth through modulation of apoptosis- and angiogenesis-related pathways. ↑ Upregulation, ↓ Downregulation.

This study demonstrates the multifaceted oncogenic role of NRF2 in KURAMOCHI ALDH^+^ ovarian cancer stem-like cells. NRF2 overexpression activated ERK and AKT signaling and enhanced proliferative behavior, while markedly enhancing cell migration and invasion. In ALDH^+^ cells, NRF2 conferred significant resistance to paclitaxel and carboplatin, accompanied by upregulation of the expression of anti-apoptotic proteins and suppression of caspase-3 activation. Consistent with these in vitro observations, NRF2-overexpressing cells exhibited enhanced tumor growth in vivo, along with increased expression of antioxidant and angiogenic factors. Together, these results indicate that NRF2 promotes aggressive phenotypes, chemoresistance, and tumorigenicity by coordinating the regulation of survival, motility, and stress-response pathways in ovarian cancer stem-like cells (Figure 5).

The ALDH^+^ KURAMOCHI cell line was selected as the experimental model because KURAMOCHI cells are a well-established high-grade serous ovarian cancer model that closely resembles the molecular characteristics of patient tumors [16], and the ALDH^+^ subpopulation is enriched for cancer stem-like cells with enhanced tumor-initiating capacity [17], making it a relevant system for investigating the role of NRF2 in regulating tumor cell proliferation and stemness-associated signaling.

In this study, NRF2 increased cancer cell proliferation both in vitro and in vivo. The previous study has shown NRF2 is activated by upstream factors, such as CEBPB, which increase its activity and directly upregulate DUSP1 transcription [18]. This, in turn, modulates the MAPK pathway [18]. Specifically, this increases ERK1/2 phosphorylation and decreases JNK and p38 phosphorylation, thereby facilitating the proliferation and survival of ovarian cancer cells [18]. NRF2 also induces the expression of antioxidant enzymes, including HO-1, CAT, GPx, and SOD. These enzymes protect cancer cells from oxidative stress and contribute to chemoresistance by neutralizing drug-induced reactive oxygen species (ROS) [12]. Persistent or aberrant NRF2 activation in ovarian cancer cells is associated with tumor progression, metabolic reprogramming, and resistance to chemotherapy [19,20]. Contrastingly, the inhibition of NRF2 signaling results in cell cycle arrest and reduced tumor growth, highlighting its role in malignant proliferation [21]. Consistent with these findings, NRF2 overexpression promoted the survival, proliferation, and chemoresistance of KURAMOCHI ALDH^+^ cells in the present study.

Interestingly, although both KURAMOCHI ALDH^+^-LENTI and KURAMOCHI ALDH^+^-NRF2 cells demonstrated enhanced wound closure compared with parental controls, the migration area at 6 h was higher in the KURAMOCHI ALDH^+^-LENTI group than in the NRF2-overexpressing group. This discrepancy may reflect the multifactorial nature of the wound-healing assay, which captures not only directional cell migration but also short-term proliferative activity and collective cell movement. Lentiviral transduction itself may transiently activate stress-responsive or cytoskeletal remodeling pathways, modestly enhancing early motility [22]. In contrast, NRF2 overexpression, while promoting invasive behavior as confirmed by the Transwell assay, may induce broader transcriptional reprogramming related to oxidative stress adaptation and metabolic regulation, potentially modulating the balance between proliferation and migration during early wound closure. These findings suggest that NRF2 may have a more pronounced effect on three-dimensional invasion rather than on short-term two-dimensional lateral migration.

Interestingly, although NRF2 overexpression enhanced migratory and invasive capacity in vitro, the tumor size in the KURAMOCHI ALDH^+^-LENTI group was larger than that in the KURAMOCHI ALDH^+^-NRF2 group in the xenograft model. This apparent discrepancy may reflect the context-dependent role of NRF2 in tumor biology. While NRF2 activation promotes oxidative stress adaptation and invasive behavior [23], it does not necessarily translate into accelerated tumor mass expansion in vivo. Tumor growth in xenograft models is influenced by multiple factors, including clonal selection during lentiviral transduction, microenvironmental stress, angiogenesis, metabolic adaptation, and host–tumor interactions [24]. NRF2 overexpression may preferentially enhance cellular survival under oxidative stress rather than directly increasing proliferative rate, thereby uncoupling invasive potential from overall tumor volume [25]. These findings highlight the complex and environment-dependent functions of NRF2 in ovarian cancer progression.

NRF2 increases HO-1 expression in ovarian cancer cells [12]. Upon activation by oxidative stress, this transcription factor translocates to the nucleus and binds to antioxidant response elements in the promoter regions of target genes, including HO-1 [26]. NRF2 enhances cellular antioxidant defense and contributes to chemoresistance by upregulating HO-1 expression in ovarian cancer cells [15]. This mechanism has been observed in both healthy and malignant ovarian epithelial cells. It is part of a broader cytoprotective response that includes other antioxidant enzymes. The NRF2–HO-1 axis is frequently upregulated in ovarian cancer. This axis is associated with tumor progression, increased survival under oxidative stress, and resistance to therapy [27]. Thus, our findings also align with previous studies showing that NRF2 overexpression increases HO-1 expression in tumor tissues.

NRF2 increases VEGFA expression in ovarian cancer cells [28]. This effect is mediated through a pathway in which follicle-stimulating hormone (FSH) induces ROS production and activates NRF2 signaling [28]. NRF2 activation subsequently promotes VEGFA expression, in part by facilitating hypoxia-inducible factor 1α signaling and its binding to the VEGFA promoter. NRF2 knockdown or ROS elimination blocks FSH-induced upregulation of VEGFA expression, demonstrating the importance of NRF2 during this process in human epithelial ovarian cancer cells [28]. Additionally, the interplay between NRF2 and VEGFA is supported by broader evidence in cancer biology. NRF2 activation contributes to angiogenesis by upregulating VEGFA expression, and microRNAs may co-regulate both NRF2 and VEGFA in ovarian cancer [29]. This regulatory axis is relevant to tumor progression and angiogenesis in ovarian cancer. Consistent with these findings, NRF2 overexpression in KURAMOCHI ALDH^+^ cells upregulated VEGFA expression in our study. This study provides a potential therapeutic target—NRF2-mediated stress response signaling—against ovarian cancer. Targeting this process could prevent chemoresistance and improve outcomes in patients with ovarian cancer.

This study is limited by the use of a single ovarian cancer cell line and gain-of-function NRF2 overexpression models, which may not fully capture ovarian cancer heterogeneity or the effects of endogenous NRF2 dysregulation in clinical settings.

## 4. Materials and Methods

### 4.1. Ethics Consideration

The study protocol was approved by the Research Ethics Committee of Hualien Tzu Chi Hospital (IRB Number: IRB112-221-C).

### 4.2. Culture of Ovarian Cancer Cells

KURAMOCHI cells were purchased from the American Type Culture Collection (Manassas, VA, USA). These cells were cultured in DMEM/F12 (Sigma-Aldrich, St. Louis, MO, USA) supplemented with 10% fetal bovine serum (Biological Ind., Kibbutz, Israel) and 1% penicillin/streptomycin (Sigma-Aldrich) at 37 °C in a 5% CO_2_ humidified atmosphere. ALDH^+^ KURAMOCHI cells were sorted via flow cytometry, identified as cancer stem cell-like populations, and subsequently utilized for downstream assays.

### 4.3. NRF2 Overexpression

NRF2 lentivectors (human, cytomegalovirus (CMV); pLenti-GIII-CMV-GFP-2A-Puro) or empty controls were purchased from ABM Company (Richmond, BC, Canada). Transfection was performed according to the manufacturer’s instructions. A multiplicity of infection of five viral particles per cell was used. KURAMOCHI ALDH^+^ cells transfected with NRF2 constructs are referred to as KURAMOCHI ALDH^+^-NRF2. Cells transfected with the empty control vector are denoted as KURAMOCHI-lenti-ctrl. Cancer cells were infected twice with lentiviruses and selected in the presence of puromycin. The resulting cells were maintained under original cell culture conditions and compared with the original cancer cells.

### 4.4. qPCR Analysis of NRF2-Overexpressing Cancer Cells

A PureLink RNA Mini Kit (Life Technologies, Carlsbad, CA, USA) was used to purify total RNA. SuperScript III enzyme (Invitrogen, Carlsbad, CA, USA) was used to reverse transcribe 1 μg of total RNA into cDNA. The Fast SYBR Green Master Mix (Applied Biosystems, Foster City, CA, USA) was used for real-time polymerase chain reaction (qPCR), and Quant Studio 5 version 3.0 (Applied Biosystems) software was used to analyze the data. Actin was used as an internal control. All experiments were conducted in triplicate. The primer sequences for the genes of interest are listed in Table 2.

### 4.5. Cell Proliferation

The XTT assay (Biological Industries Ltd., Beit Haemek, Israel) was performed to investigate the effect of NRF2 overexpression on cell proliferation. Ovarian cancer cells, with or without NRF2 overexpression, were seeded in 96-well plates at a density of 2000 cells/cm^2^. Cell proliferation was evaluated on days 0, 3, and 5. Day 5 was selected as the final experimental time point because cells approached near-confluence beyond this stage under the culture conditions, which could affect the accuracy of proliferation measurements. For the XTT assay, solutions of XTT and N-methyl dibenzopyrazine methyl sulfate (PMS; Biological Industries Ltd.) were thawed at 37 °C immediately before use. PMS was mixed with the XTT reagent immediately before application, and 50 μL of the XTT/PMS mixture was added to each 100 μL of culture medium. After incubation for 2–5 h at 37 °C, absorbance was measured at 450 nm with a reference wavelength of 650 nm using a microplate spectrophotometer (DYNEX MRX II; Dynex Technologies, Chantilly, VA, USA). All experiments were performed in triplicate.

### 4.6. Western Blot Analysis of NRF2, Cell Proliferation-Related Proteins, and Apoptotic-Related Proteins

Ovarian cancer cells (1 × 10^6^) (KURAMOCHI ALDH^+^, KURAMOCH-ALDH^+^-lenti-ctrl, and KURAMOCHI ALDH^+^-NRF2) were cultured in a 10 cm culture dish for 24 h. A lysis buffer (150 mM NaCl; 50 mM Tris–HCl, pH 7.4; 1% Nonidet P-40) and a proteinase inhibitor cocktail (04693116001; Roche, Basel, Switzerland) were used to lyse the tested cells. Furthermore, 10% sodium dodecyl sulfate–polyacrylamide gel electrophoresis was performed, and the gel was transferred to a nitrocellulose membrane (Hybond-C Super; GE Healthcare, Little Chalfont, UK). The antibody employed was NRF2 (1:1000, A1244; ABclonal, Woburn, MA, USA). Actin (#4970; Cell Signaling Technology, Danvers, MA, USA) was used as an internal control. Membranes were incubated with the antibodies described above. A horseradish peroxidase-conjugated goat anti-mouse IgG secondary antibody (AS003; Abclonal) was used to bind the primary antibody. Enhanced chemiluminescence reagents (GE Healthcare) were used to detect the bound antibodies.

The cell proliferation and apoptotic pathways were also evaluated. The roles of AKT (#9272, phospho-AKT, #9271; Cell Signaling Technology) and ERK (#4695, phospho-ERK, #4270; Cell Signaling Technology) in cell growth signaling were assessed. Additionally, BCL2 (#12789-1-AP; Proteintech, Chicago, IL, USA), X-linked inhibitor of apoptosis (XIAP, ab21278; Abcam, Cambridge, UK), and cleaved caspase-3 (#9664; Cell Signaling Technology) were used to examine apoptotic signaling. Protein quantification data were normalized to the total protein loading (actin). Relative quantification was then compared with that of the original cells for fold-change analysis.

### 4.7. Wound-Healing Assay

A wound-healing (scratch) assay was performed to investigate the effects of NRF2 overexpression on cell migration. KURAMOCHI ALDH^+^ ovarian cancer cells, with or without NRF2 overexpression, were seeded into 6-well plates at a density of 3 × 10^5^ cells/well and cultured until a confluent monolayer was formed. A uniform scratch was created across the monolayer using a sterile 200 μL pipette tip to generate a wound gap. The detached cells were gently removed by washing with PBS, and the cultures were incubated in fresh medium. Images of the wound area were captured at 0 (immediately after scratching) and 6 h using phase-contrast microscopy. The wound area was quantified using ImageJ software version 1.54p, and the percentage of wound closure was calculated using the following formula: [Wound closure (%) = (Area_{0 h} − Area_{6 h})/Area_{0 h} × 100]. All experiments were performed in triplicate.

### 4.8. Transwell Invasion Assay

A Transwell invasion assay was performed to investigate the effect of NRF2 overexpression on cell invasion. KURAMOCHI ALDH^+^ ovarian cancer cells with or without NRF2 overexpression (5 × 10^4^ cells) were seeded onto Matrigel-coated Transwell inserts in 24-well plates (BD BioCoat Matrigel Invasion Chamber; BD Biosciences, Bedford, MA, USA). The lower chamber was filled with culture medium supplemented with regular medium. After 24 h of incubation, the non-invading cells and residual Matrigel on the upper surface of the membrane were carefully removed using a cotton-tipped applicator. This process was repeated using a fresh applicator to ensure complete removal. Invading cells that adhered to the underside of the membrane were fixed with methanol for 15 min and stained with 0.1% crystal violet for 20 min at room temperature. Excess dye was removed by rinsing with distilled water, and the membranes were air-dried before mounting onto slides. Invading cells were visualized under a light microscope, and the number of stained cells was counted in three randomly selected fields per insert. The mean number of invading cells per field was calculated, and all experiments were performed in triplicate.

### 4.9. Evaluation of Chemosensitivity

The IC_50_ assay was used to assess the effect of NRF2 overexpression on cancer cell chemosensitivity. Paclitaxel (Formoxol; Yung Shin Pharm. Ind., Co., Ltd., Taichung, Taiwan) and carboplatin (SINPHAR Pharmaceutical Co., Ltd., Yilan, Taiwan), two common chemotherapeutic agents used to treat ovarian cancer, were investigated in ovarian cancer cells with or without NRF2 overexpression. Cells (3 × 10^3^/well) were seeded in a 96-well plate and treated with increasing concentrations of the chemotherapeutic drugs for 48 h to determine their IC_50_ values. Cell concentration after chemotherapy was measured by adding XTT solution and recording the optical density. The IC50 values were calculated using a nonlinear regression model in GraphPad Prism (version 9.0; GraphPad Software, San Diego, CA, USA).

### 4.10. Animal Xenograft Experiment

An in vivo tumor-formation assay was performed to investigate the effect of NRF2 overexpression on tumor growth. KURAMOCHI ALDH^+^ cancer stem cells with or without NRF2 overexpression were used. All animal experiments were approved by the Animal Research and Care Committee of Hualien Tzu Chi Hospital. NOD-SCID mice (strain: NOD.CB17-Prkdcscid/JTcu, 5-week-old) were provided by Tzu Chi University. Ten mice were used for experiments. KURAMOCHI ALDH^+^ ovarian cancer cells with or without NRF2 overexpression (1 × 10^5^ cells) were injected into the subcutaneous tissue of the dorsal region (*n* = 3 in the control and lenti-ctrl groups, *n* = 4 in the NRF2 group). Before transplantation, KURAMOCHI ALDH^+^ cells were cultured for seven days to obtain the required cell numbers. For injection, cells were suspended in 100 μL of culture medium and mixed with 100 μL of growth factor-reduced Matrigel (BD Matrigel™ Matrix; BD Biosciences). Mice were anesthetized with 2% isoflurane, and oxygen was delivered at 2 L/min during the procedure. Mice were housed in pathogen-free rooms at the Animal Center of Tzu Chi University. The relative humidity was maintained at 45–65%, and cages were maintained at 20–24 °C.

The mice were euthanized through CO_2_ exposure once the tumor size reached approximately 500 mm^3^. The tumors were excised, photographed, measured, and weighed. Tumor growth was monitored weekly for one–two months, and tumor volumes were calculated using the following formula: (width^2^ × length)/2.

### 4.11. Histological Examination

Xenograft tumor tissues were fixed in 4% paraformaldehyde and sectioned at 4 μm. Hematoxylin and eosin staining was performed for histological examination. Tumor sections were viewed at 200× magnification. Nuclear morphology, cell density, and mitotic figures were recorded.

### 4.12. Western Blot Analysis of Xenograft Tumor Tissue

The tumor tissues were harvested, rinsed with cold PBS, and divided for downstream applications. For Western blotting, approximately 20–50 mg of tissue was homogenized in ice-cold RIPA lysis buffer supplemented with protease and phosphatase inhibitors.

The homogenates were incubated on ice for 30 min with intermittent vortexing and then centrifuged at 14,000× *g* for 15 min at 4 °C to remove insoluble debris. The resulting supernatants were collected as clarified protein lysates. Protein concentrations were measured using the BCA assay, and aliquots were stored at −80 °C until use.

Tumor lysates were analyzed for HO-1 (anti-oxidative stress enzyme) and VEGF-A (pro-angiogenesis); β-Actin served as the loading control (all from Cell Signaling). Band intensities were quantified by densitometry (ImageJ). The background was subtracted, each target band was normalized to its corresponding β-actin band and values were expressed relative to the control lane (set to 1.0). Densitometric values normalized to the control are shown below each blot band.

### 4.13. Statistical Analysis

Student’s *t*-test was used to compare continuous variables between two groups. Data are presented as the mean and standard deviation. The Chi-square test was used to assess categorical variables. Analysis of variance with a post hoc Bonferroni test was used to analyze data derived from more than two groups. Statistical analyses were performed using SPSS software (version 24, IBM, Armonk, NY, USA). Statistical significance was set at *p* < 0.05.

## 5. Conclusions

This study demonstrated that NRF2 exerts a context-dependent yet predominantly oncogenic role in ovarian cancer stem-like cells. NRF2 overexpression enhances ERK and AKT signaling, promotes migratory and invasive behavior, and confers resistance to platinum- and taxane-based chemotherapy by suppressing apoptosis. NRF2 markedly increases tumor growth in vivo, accompanied by the upregulation of antioxidant and angiogenic pathways. These findings highlight NRF2’s regulatory role in ovarian cancer aggressiveness and therapeutic resistance, suggesting that targeting NRF2-mediated stress response signaling is a promising strategy to overcome chemoresistance and may improve outcomes in patients with ovarian cancer.

## Figures and Tables

**Table 1:** Semi-quantitative histopathological scoring of xenograft tumors derived from KURAMOCHI ALDH^+^ cells.

Histopathological Feature	Control (*n* = 3)	Lenti-Control (*n* = 3)	NRF2 Overexpression (*n* = 4)	*p*-Value
Tumor cell density	1.0 ± 0.0	2.0 ± 0.0	3.0 ± 0.0	<0.01
Nuclear enlargement	1.0 ± 0.0	2.0 ± 0.0	3.0 ± 0.0	<0.01
Nuclear pleomorphism	1.0 ± 0.0	2.0 ± 0.0	3.0 ± 0.0	<0.01
Nuclear hyperchromasia	1.0 ± 0.0	2.0 ± 0.0	3.0 ± 0.0	<0.01
Mitotic figures	0.5 ± 0.5	1.5 ± 0.5	2.5 ± 0.5	<0.01
Tumor architecture compactness	1.0 ± 0.0	2.0 ± 0.0	3.0 ± 0.0	<0.01
Overall pathology score	0.9 ± 0.2	1.9 ± 0.3	2.9 ± 0.3	<0.001

Scoring scale: 0 = absent, 1 = mild, 2 = moderate, 3 = marked. Data are presented as mean ± SD.

**Table 2:** Primer sequences of candidate genes.

Gene	Forward Sequence (5′–3′)	Reverse Sequence (5′–3′)	Product Size (bp)
*NRF2*	CACATCCAGTCAGAAACCAGTGG	GGAATGTCTGCGCCAAAAGCTG	112
*Actin*	CACCATTGGCAATGAGCGGTTC	AGGTCTTTGCGGATGTCCACGT	135

## Data Availability

The original contributions presented in this study are included in the article/Supplementary Material. Further inquiries can be directed to the corresponding author.

## Supplementary Materials

The original blot images can be downloaded at: https://www.mdpi.com/article/10.3390/ijms27062820/s1.

## Abbreviations

The following abbreviations are used in this manuscript:

NRF2	Nuclear factor erythroid-derived 2–like 2
ERK	Extracellular signal-regulated kinase
ROS	Reactive oxygen species
FSH	Follicle-stimulating hormone
PMS	N-methyl dibenzopyrazine methyl sulfate
BCL2	B cell lymphoma 2
XIAP	X-linked inhibitor of apoptosis
IC_50_	Half-maximal inhibitory concentration
NOD-SCID	Non-obese, diabetic-severe combined immune deficiency
ALDH	Acetaldehyde dehydrogenase
