# Supplementary material for: NRF2 Drives Aggressiveness and Chemoresistance in Ovarian Cancer Stem-like Cells
Source: Int J Mol Sci. 2026 Mar 20;27(6):2820. doi: 10.3390/ijms27062820 (PMC13026881; doi:10.3390/ijms27062820)
Supplement: Supplementary file 1 [file ijms-27-02820-s001.zip › ijms-4164243-supplementary.pdf]

Original blot

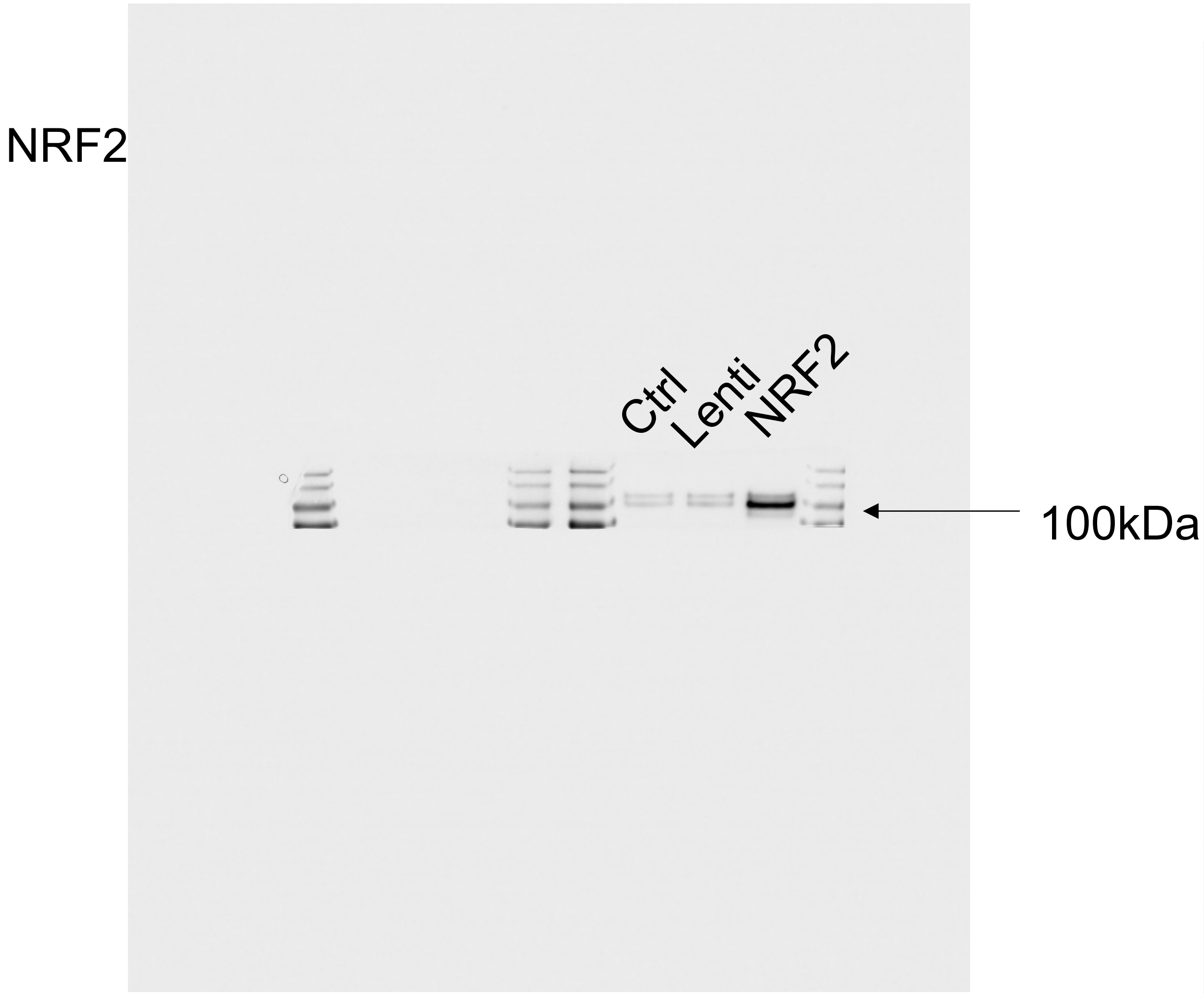
NRF2
Ctrl
Lenti
NRF2
100kDa

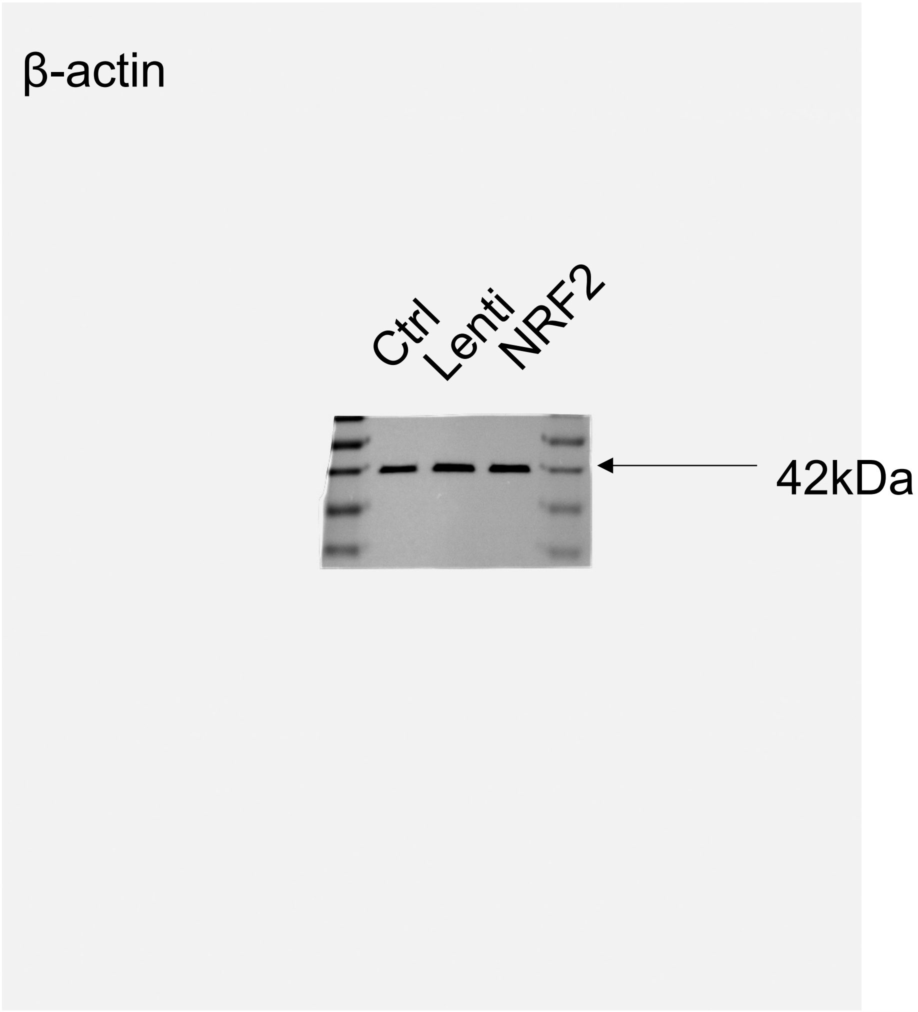
β-actin
Ctrl
Lenti
NRF2
42kDa

ERK

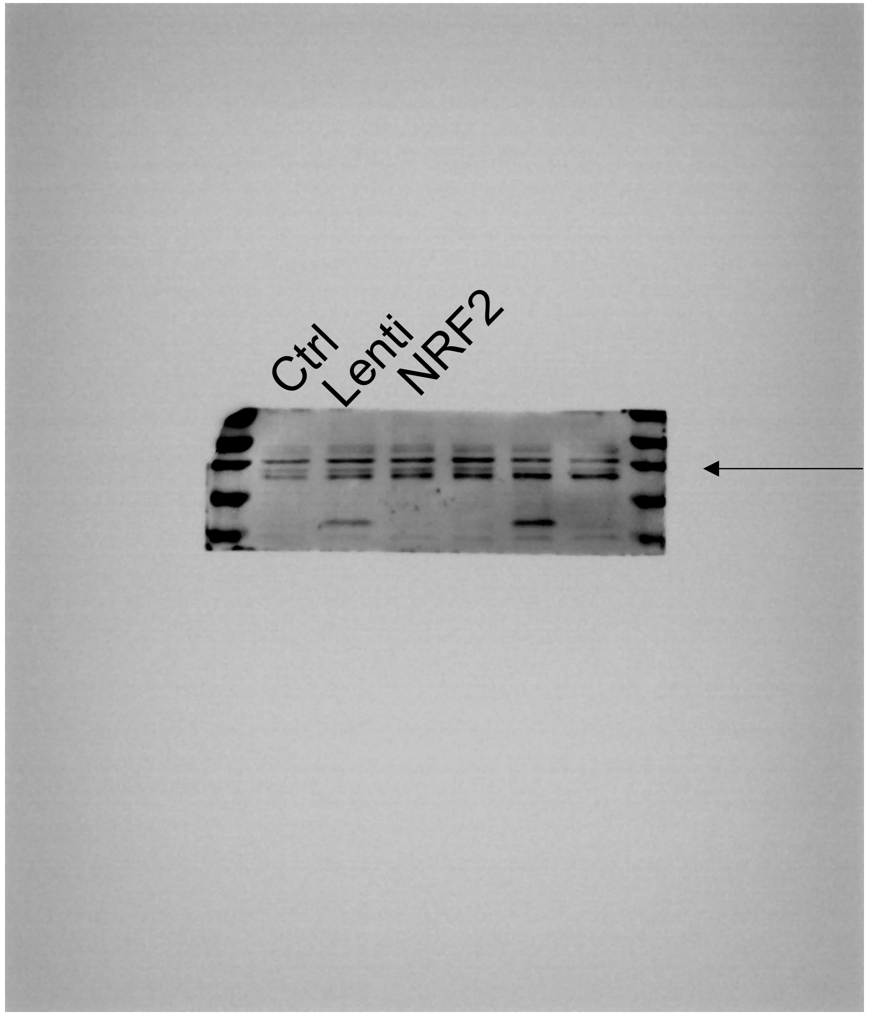

42-44kDa

p-ERK

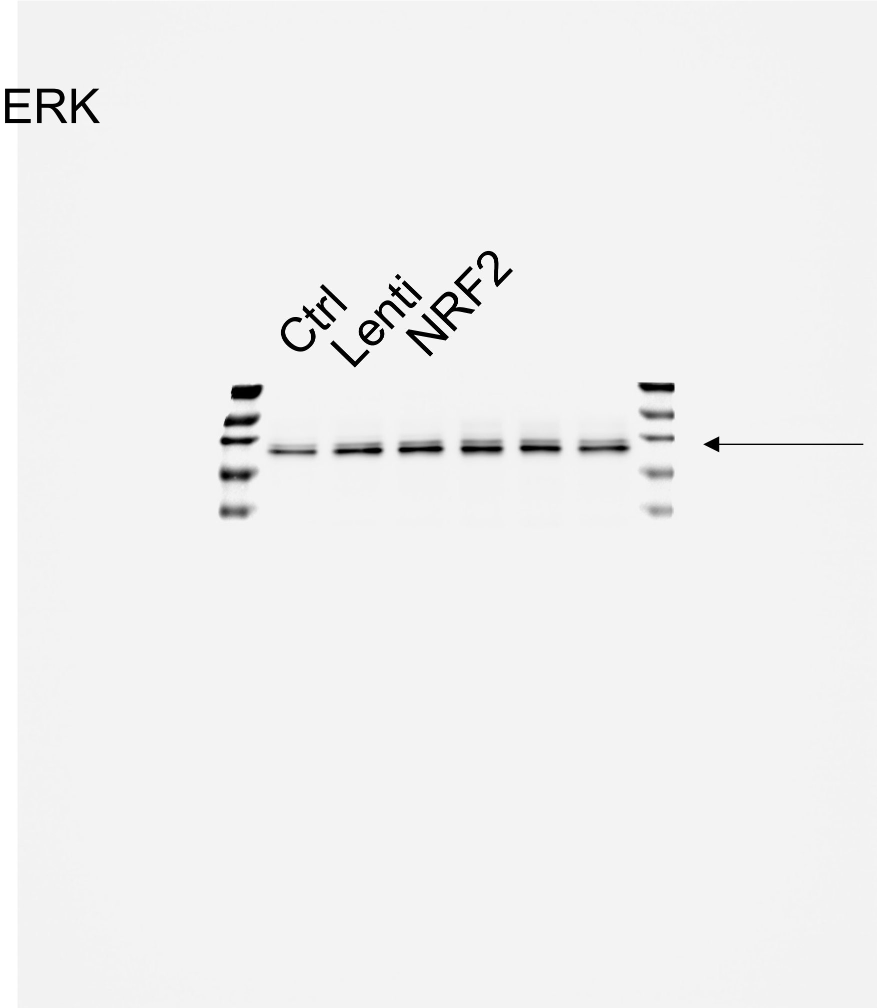

44kDa

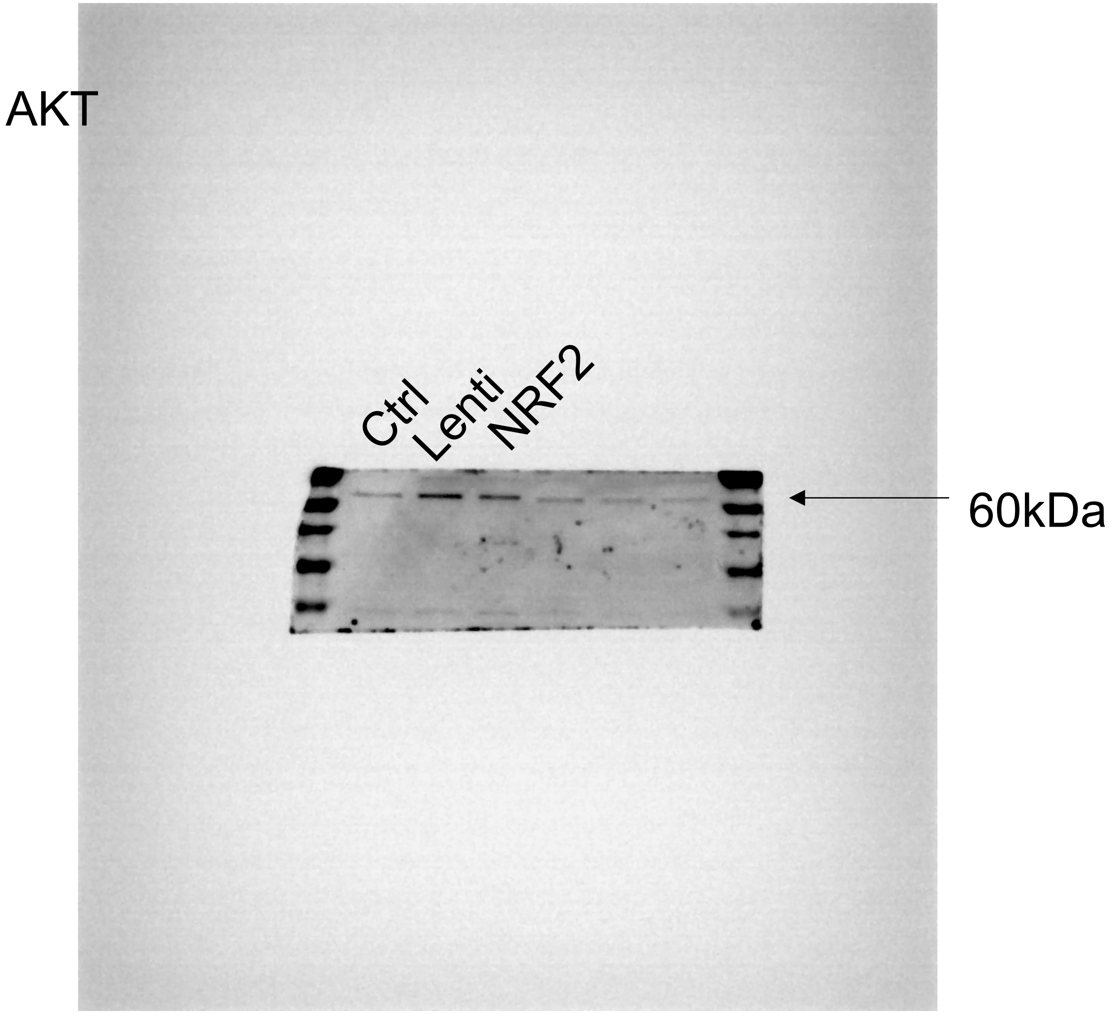
AKT
Ctrl
Lenti
NRF2
60kDa

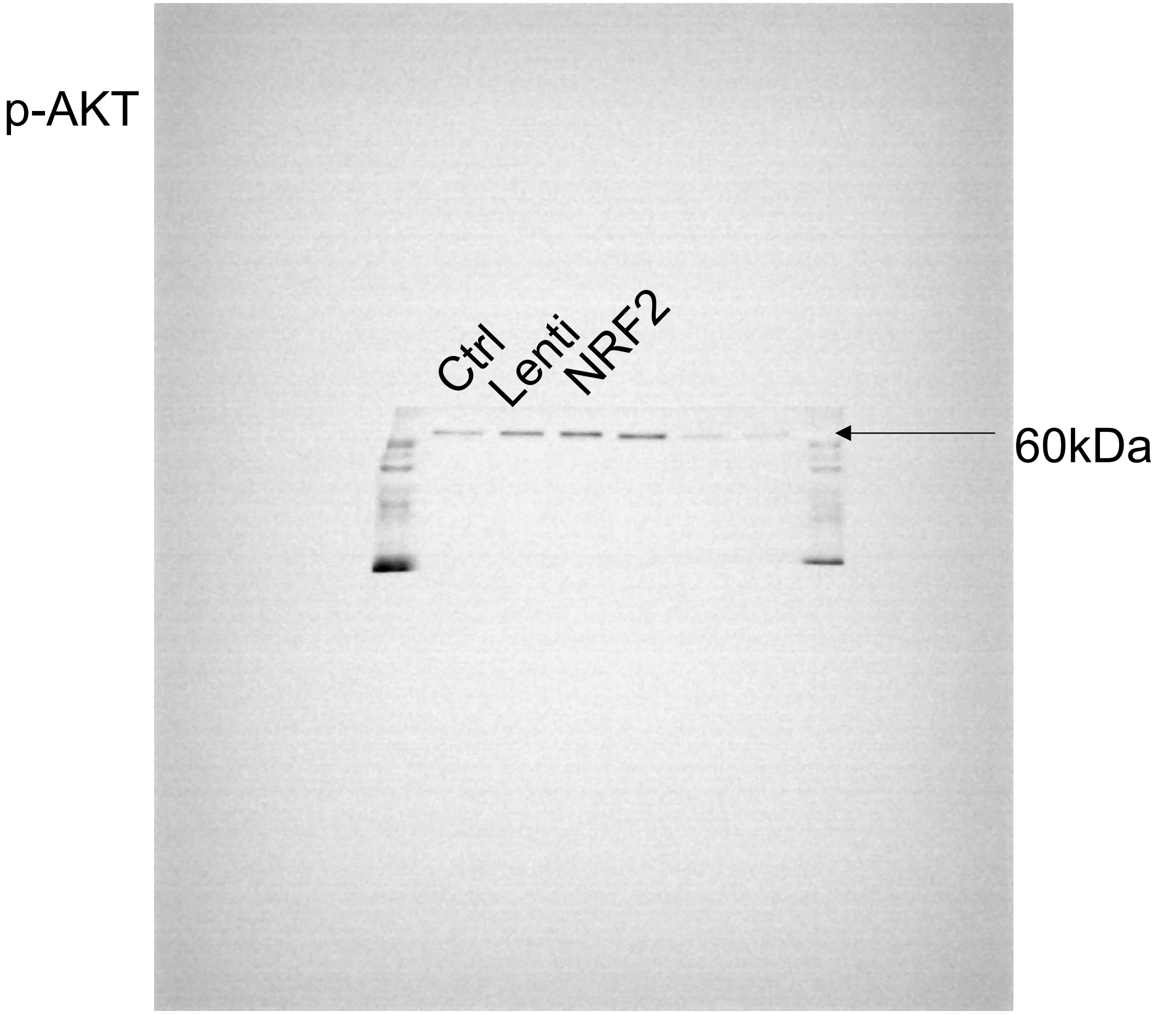
p-AKT
Ctrl
Lenti
NRF2
60kDa

BCL2

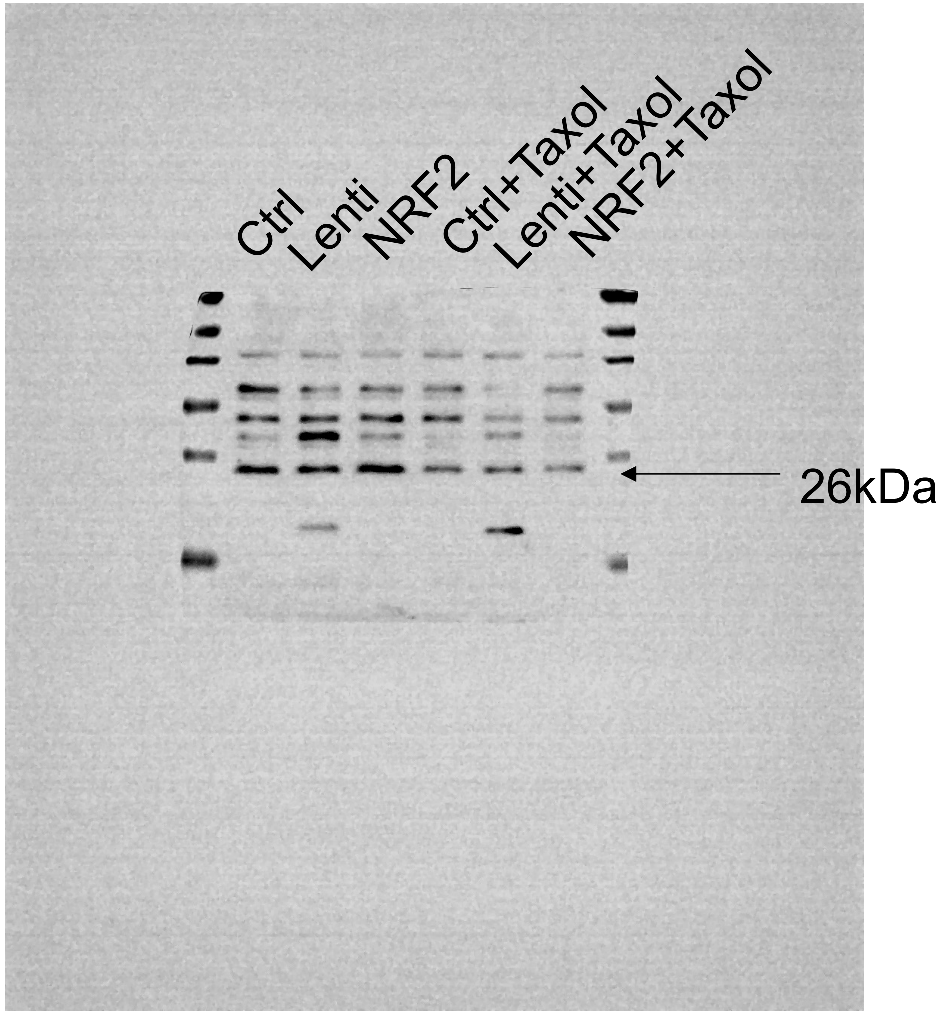
Ctrl
Lenti
NRF2
Ctrl+Taxol
Lenti+Taxol
NRF2+Taxol
26kDa

β-actin

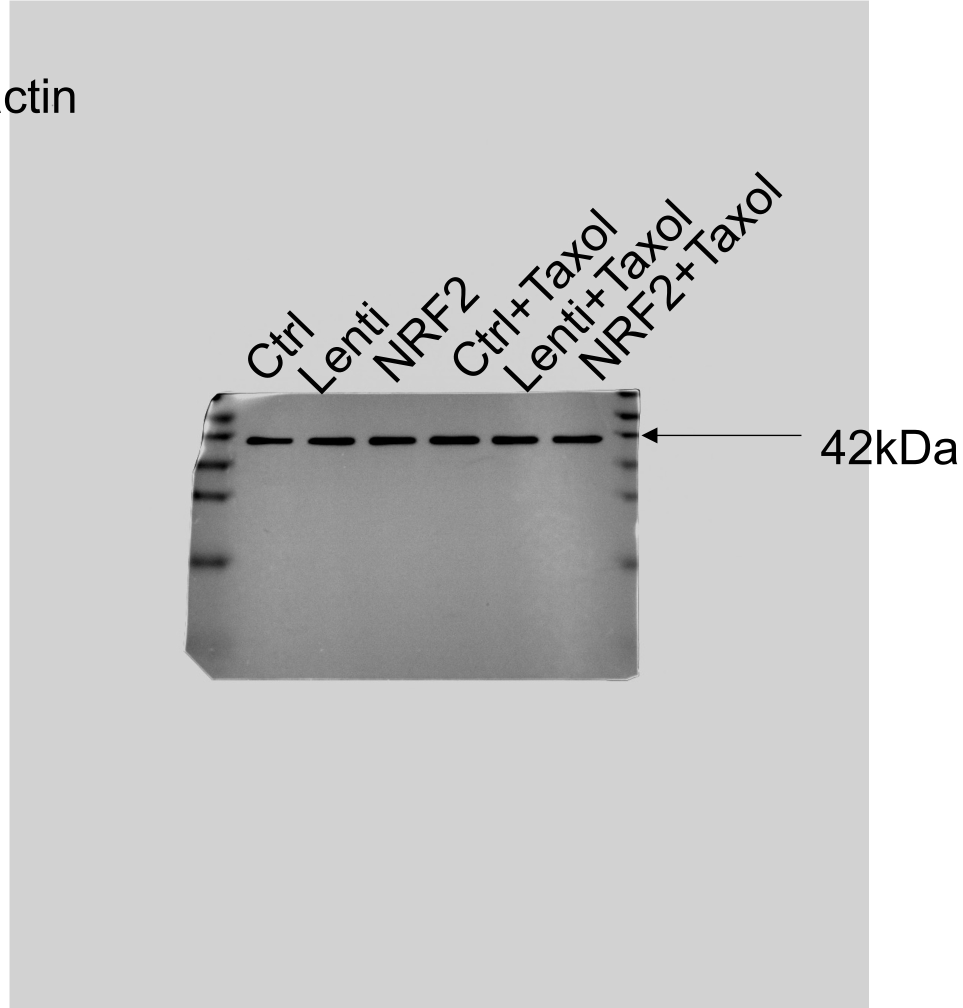
Ctrl
Lenti
NRF2
Ctrl+Taxol
Lenti+Taxol
NRF2+Taxol
42kDa

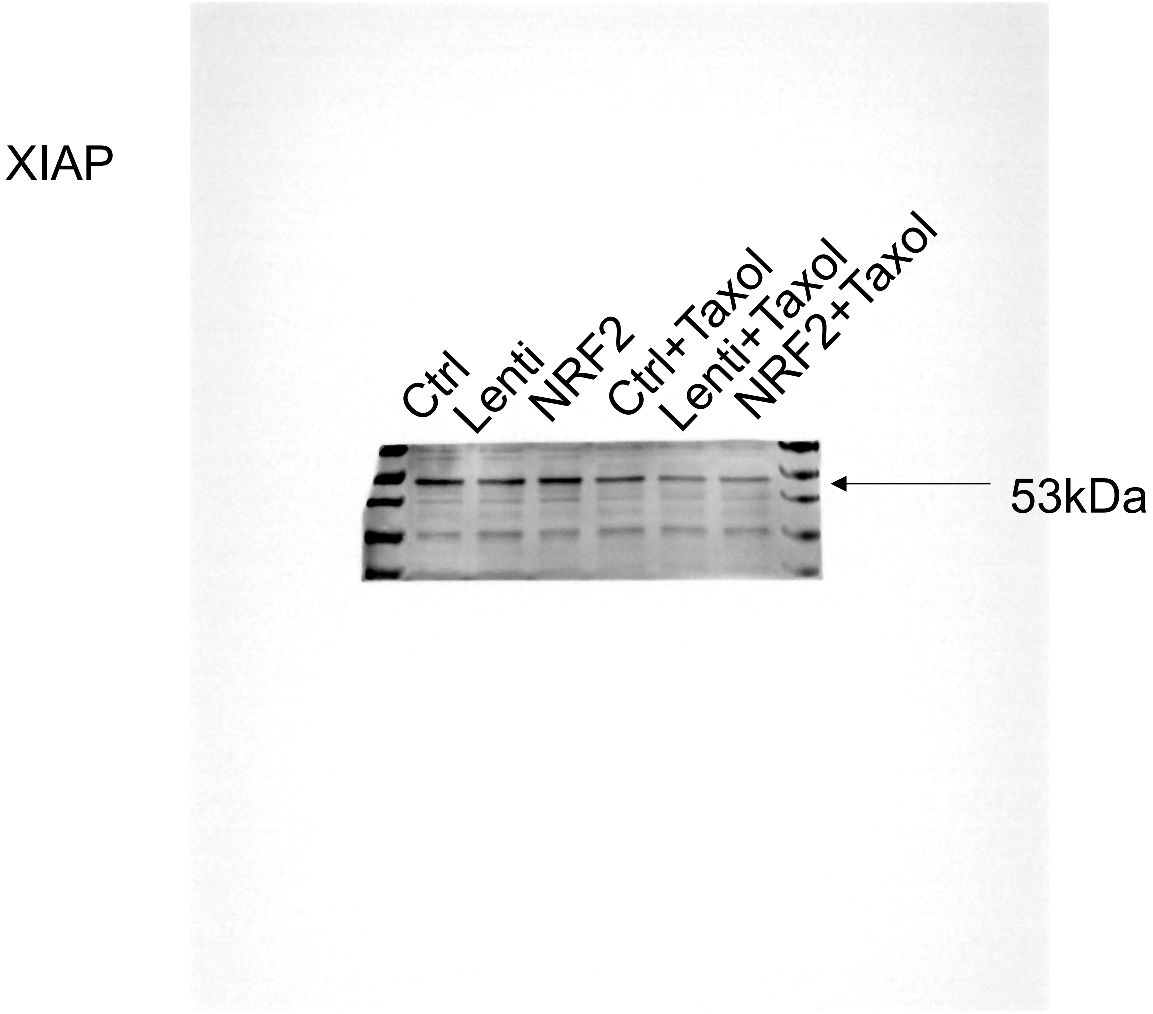
XIAP
Ctrl
Lenti
NRF2
Ctrl+Taxol
Lenti+Taxol
NRF2+Taxol
53kDa

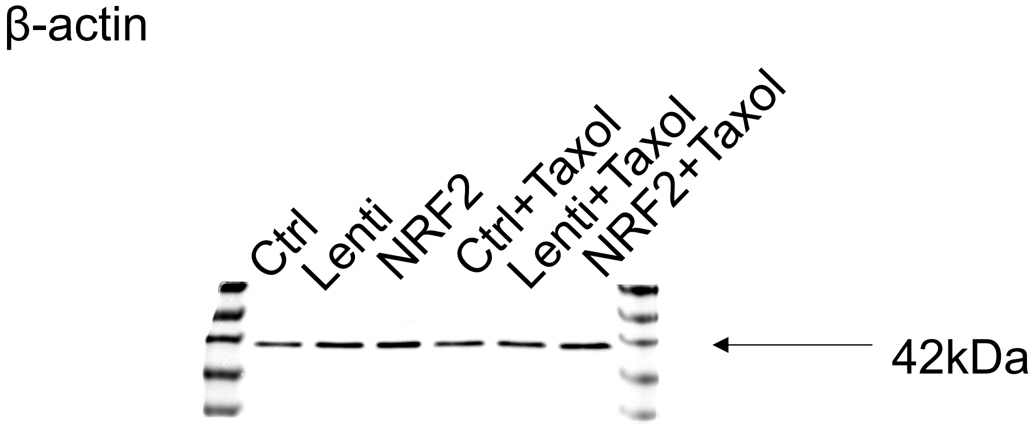
β-actin
Ctrl
Lenti
NRF2
Ctrl+Taxol
Lenti+Taxol
NRF2+Taxol
42kDa

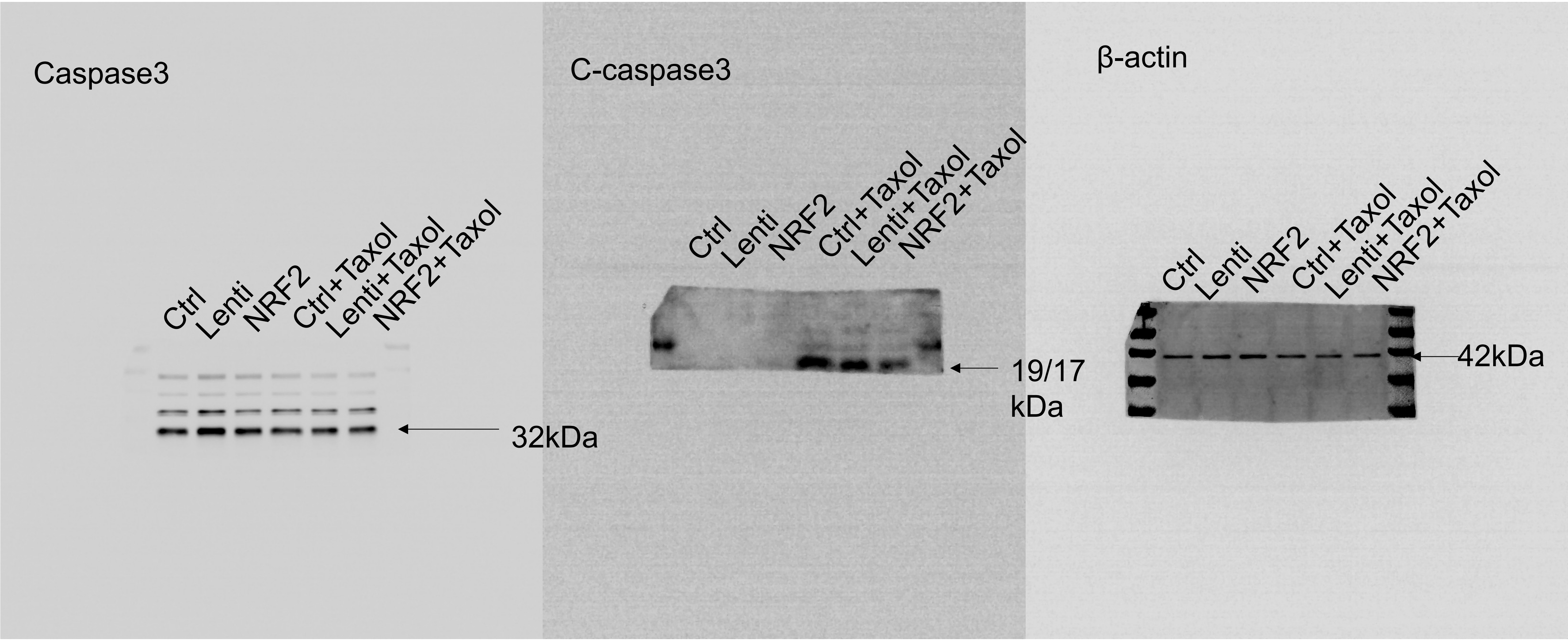
Caspase3
Ctrl
Lenti
NRF2
Ctrl+Taxol
Lenti+Taxol
NRF2+Taxol
32kDa
C-caspase3
Ctrl
Lenti
NRF2
Ctrl+Taxol
Lenti+Taxol
NRF2+Taxol
19/17
kDa
β-actin
Ctrl
Lenti
NRF2
Ctrl+Taxol
Lenti+Taxol
NRF2+Taxol
42kDa

HO-1

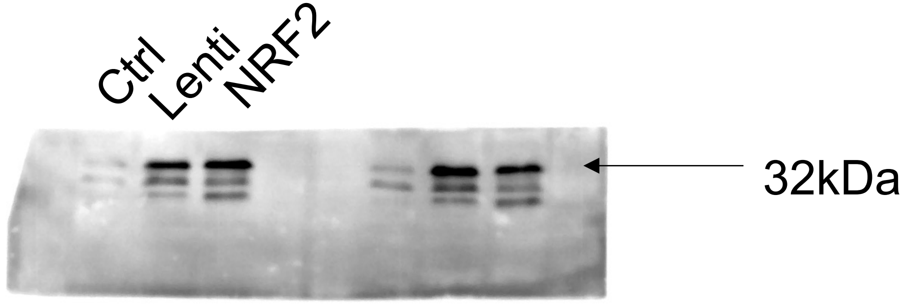

β-actin

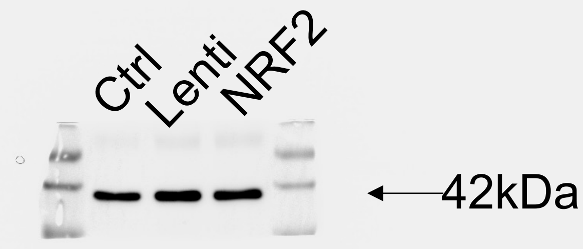

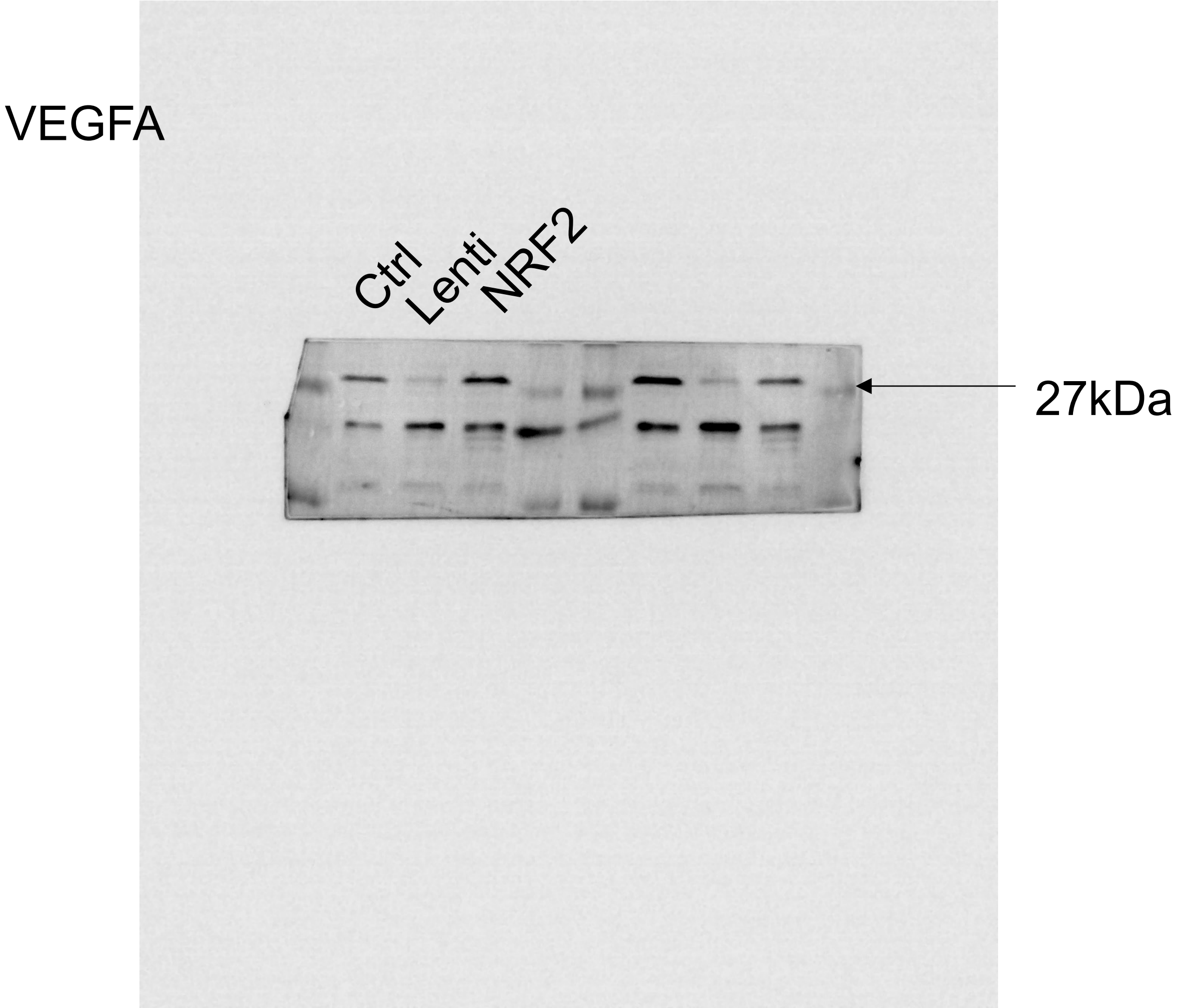

VEGFA
Ctrl
Lenti
NRF2
27kDa

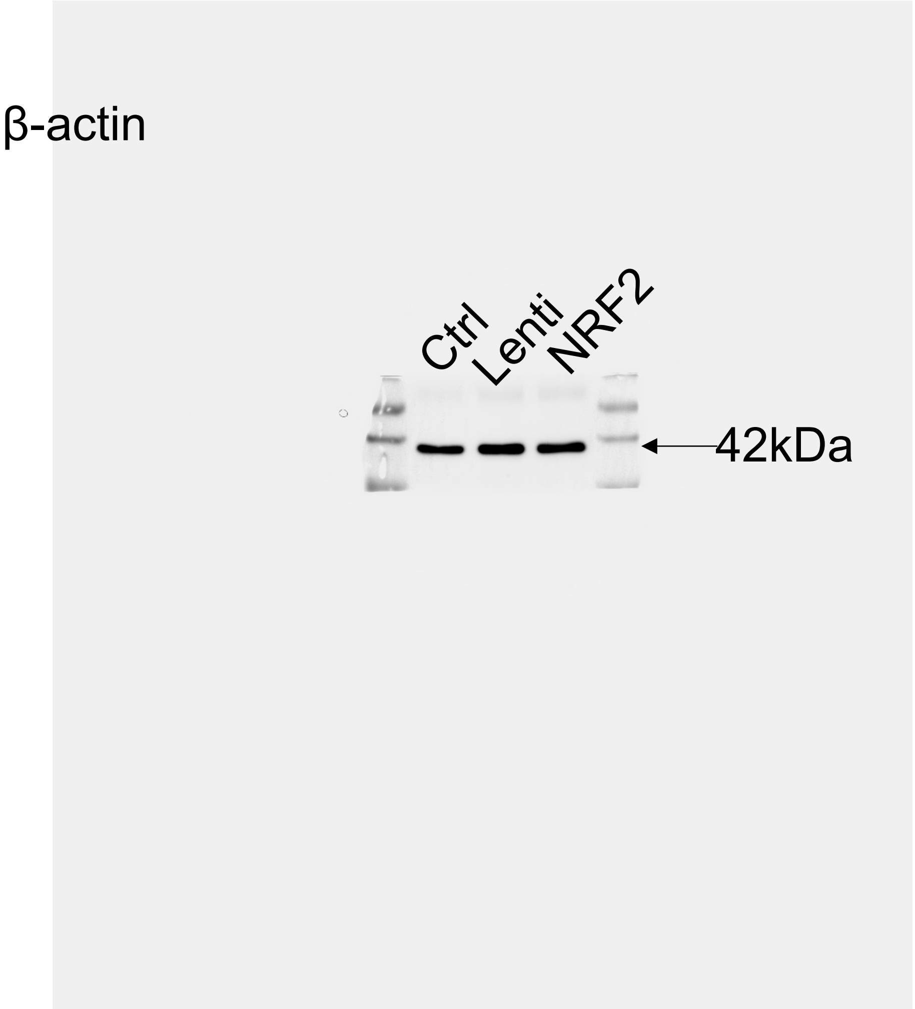

β-actin
Ctrl
Lenti
NRF2
42kDa
